# Brachial muscle injury resulting in acute compartment syndrome of the upper arm: a case report and literature review

**DOI:** 10.1186/s12891-021-04318-1

**Published:** 2021-06-14

**Authors:** Lei Tan, Yongning Xia, Zilong Su, Qiangqiang Wen, Jiting Zhang, Tiecheng Yu

**Affiliations:** Department of Orthopedic Trauma, The First Hospital of Jilin University, No. 71 Xinmin Street, Changchun, 130021 Jilin China

**Keywords:** Acute compartment syndrome, Upper arm, Fasciotomy, Brachial muscle

## Abstract

**Background:**

Acute compartment syndrome (ACS) is a potentially devastating condition. ACS is rare in the upper arm.

**Case presentation:**

We report a case of acute compartment syndrome of the anterior compartment of the upper arm due to brachial muscle injury. The patient experienced abnormal progressive swelling and pain in his right upper arm, and passive pulling pain of the right wrist and right hand. It was highly suspected to be right upper arm compartment syndrome, and was confirmed by surgery. The patient transferred to the emergency operating room for fasciotomy that was performed under general anesthesia using the anterolateral approach. The brachial muscle was found to be heavily swollen and had the greatest tension. The brachial muscle fibers were split lengthwise, and a large amount of hematoma was cleared. The brachial muscles were injured and partly ruptured. After full decompression, a negative pressure drainage device was used to cover the wound in the first stage. Ten days after injury, the swelling of the affected limb subsided and the wound was sutured. The patient’s limbs completely recovered to normal. The shoulder and elbow joints could move freely and the patient resumed normal farming work ability.

**Conclusion:**

Clinicians should fully recognize the fact that acute compartment syndrome can occur in the upper arm, rather than only the forearm and leg, and therefore avoid serious consequences caused by missed diagnosis and misdiagnosis.

## Introduction

Acute compartment syndrome (ACS) refers to the signs and symptoms of severe ischemia of the nerve and muscle tissue in limbs caused by increased intra- osseofascial compartment pressure. It is a potentially devastating condition that can cause ischemia, necrosis, and potentially rhabdomyolysis and death [1]. The most common causes include trauma, arterial injury, limb compression, and burns [2].

Compartment syndrome is not uncommon in the forearm and leg, but is rare in the upper arm [3]. Only a few reports have detailed this [3–6]. However, there were fewer reports of isolated anterior compartment syndrome, which were mostly caused by the injury of biceps [7–9]. The present case report will discuss acute compartment syndrome of the isolated anterior compartment of the upper arm due to brachial muscle injury after blunt trauma in a 55-year-old man. To the best of our knowledge, this is the first report of this type of injury.

## Case report

**Fig. 1:**
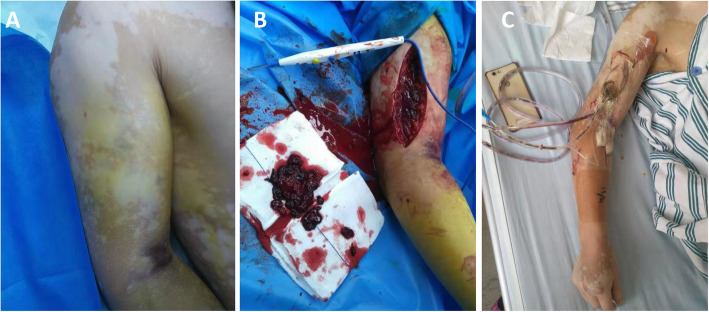
**a** Preoperative photograph showing right upper arm anterior swelling and subcutaneous ecchymosis in the elbow fossa; **b** Intraoperative photograph showing injury of the rachial muscle after fasciotomy of the anterior compartment of the upper arm and the hematoma that was cleared; **c** Picture of the treatment of the negative pressure drainage device after surgery

The institutional review board at the First Hospital of Jilin University approved this work, and the patient has provided informed consent for publication of the case. A 55-year-old farmer entered our emergency department with severe pain in the right elbow and upper arm, swelling, and partial movement limitation lasting more than 13 h. The patient had fallen when he walked up the stairs 13 h prior, and hit the handrail in the lower third of his right upper arm. Within 6 h after the injury, there were no obvious pain or swelling in the right upper arm, and no attention was paid to it. He continued his farming work. Seven hours after the injury, he went to a local hospital because of progressive swelling and pain in his right upper arm, and the local hospital transferred the patient to the emergency department of our hospital about 13 h after injury. Upon physical examination, the patient’s temperature was 37.6 C, his breathing was 18 times per minute, his pulse was 90 times per minute, and his blood pressure was 155/70 mmHg. He had a swollen, tender right elbow joint with restricted motion, right upper arm anterior swelling (the medial and lateral tension were large, especially in the front of the humerus), the size of his upper elbow fossa was about 2 cm × 1 cm with subcutaneous ecchymosis (Fig. 1a), he had pale epidermis, and an automobile tire sensation when touched on the front part of the right upper arm. The passive motion pain of the right elbow joint was positive, the pulsation of the right radial artery was weak, and the patient experienced pain when touched on the right upper arm. Numbness of the forearm was not obvious, the muscle strength of the right hand was graded 5/5, and the skin temperature was still acceptable.

The arm was subjected to radiological evaluation. Three X-ray examinations of the shoulder, humerus, and elbow of the right upper limb showed no obvious fracture. During the imaging examination, the pain symptoms were progressively aggravated, and the patient gradually lost his sensation from the elbow to the ends of the fingers. The passive pulling pain of the right wrist and right hand was positive. It was highly suspected to be right upper arm compartment syndrome.

The patient was immediately transferred to the emergency operating room for exploration and decompression under general anesthesia. A 15 cm longitudinal incision was made using the anterolateral approach (where the skin tension was the greatest) in the lower third of the right upper arm (Fig. 1b). The superficial fascia was separated and the deep fascia was opened. The pressure was not relieved, and the brachial artery was found to be intact. The tension of the posterolateral compartment and the biceps brachii muscle in the anterolateral compartment was not high, and the brachial muscle was heavily swollen and had the greatest tension. The brachial muscle fibers were split lengthwise, and a large amount of hematoma and stasis were found. The hematoma was cleared (Fig. 1b). Two small veins were broken inside the brachial muscle, and there was still non-pulsating, dark red blood outflow. Electrocoagulation was used to stop the bleeding. The brachial muscles were injured and partly ruptured. Other muscles and nerves were explored, and no obvious abnormalities were found. After full decompression, a negative pressure drainage device was used to cover the wound in the first stage (Fig. 1c). Ten days after injury, the swelling of the affected limb subsided and the wound was sutured.

**Fig. 2:**
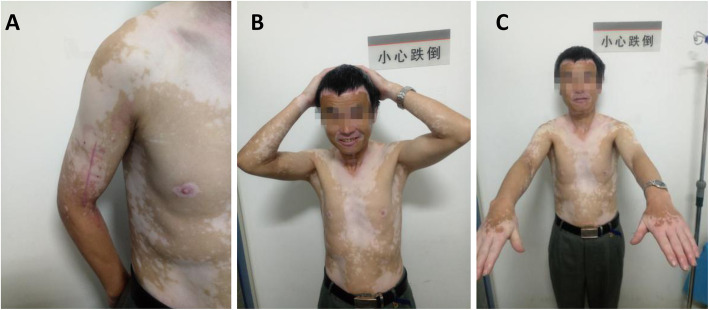
Postoperative photograph showing that the patient’s activity and function were good during the patient follow-up 40 days after surgery

The pain was relieved immediately after the operation, and the nerve sensation and motor function recovered. One week after the surgery, elbow flexing and biceps exercises were initiated. The stitches were removed 2 weeks after stitching. After 40 days of follow-up, the patient’s limb completely recovered to normal. The shoulder and elbow joints could move freely (Fig. 2), and the patient resumed normal farming work.

## Discussion and conclusion

In the existing literature on the rare reports of upper arm ACS, the following have all been reported as causes: limb compression [10], supracondylar fracture of the humerus in children [11], humeral shaft fracture [12], heroin injection poisoning [13], carbon monoxide poisoning [14], bicep muscle rupture [7, 8], triceps injury [15, 16], and severe bleeding secondary to venipuncture or thrombolytic treatment [17–19]. However, there were fewer reports of isolated anterior compartment syndrome, which were mostly caused by the injury of biceps [7–9] and secondary to drug toxicity [13, 18, 19] or fracture [11, 12]. We present a case of acute compartment syndrome of the isolated anterior compartment of the upper arm due to single brachial muscle injury after blunt trauma. To the best of our knowledge, this is the first report of this type of injury.

Only a few reports have detailed this [3–6]. However, there were fewer reports of isolated anterior compartment syndrome, which were mostly caused by the injury of biceps [7–9]. The present case report will discuss acute compartment syndrome of the isolated anterior compartment of the upper arm due to brachial muscle injury after blunt trauma in a 55-year-old man.

The injury mechanism of the presented case is rare. The force on the medial part of the upper arm only injured the brachial muscle (the deep part of the brachial muscle lies at the lower edge of the medial part of the forearm) and led to in internal hemorrhaging of the entire brachial muscle, which caused increased pressure in the compartment where the brachial muscle is located, ultimately resulting in a rare cause of upper arm compartment syndrome. In addition, the pathogenesis of the case is also very characteristic. In the early stage of the injury, no neuromuscular symptoms were found. The patient continued to engage in farm work for about 4 h after injury. However, the symptoms of the affected limbs gradually appeared and aggravated after the pressure of the upper arm fascia chamber increased. During the course of treatment, the patient was treated by splitting the extremely swollen brachial myomembrane, which also confirmed the diagnosis of single compartment syndrome in the anterior compartment of the upper right arm caused by simple brachial muscle injury.

The incidence of acute compartment syndrome in extremities is determined by its anatomical characteristics. Acute compartment syndrome is predominant in the forearm and lower leg due to the more abundant contents inside the compartments and the toughness of the interosseous and intercompartmental membranes [1]. The upper arm contains a single bone (the humerus) and no interosseous membrane. The the upper arm is divided into two compartments through the medial and lateral interventricular membrane: the anterior compartment (containing biceps, brachialis, coracobrachialis) and the posterior compartment (containing triceps). The compartment is composed of the humerus, intermuscular fascia, and fascia, which has greater flexibility and expansion space. Therefore, the incidence of upper arm compartment syndrome is very low [9].

Timely, adequate, and complete decompression is the principal treatment that must be followed for upper arm compartment syndrome. Muscles can tolerate ischemia for 4 h, but functional changes may occur after 6 h, and damage is irreversible after 8 h [5]. Once the presence of compartment syndrome of the upper arm is suspected, the reduction of tension should be carried out decisively. Any delay in diagnosis and treatment can have catastrophic consequences. Han [20] reported a case of upper arm compartment syndrome caused by blunt impact after fall, and followed by rhabdomyolysis, acute renal failure, ultimately resulting in the death of patient with intracerebral hemorrhage. Thomas [9] and Traub [3] described in detail the consequences of compartment syndrome, including permanent nerve damage, muscle necrosis, growth arrest, Volkmann muscle contracture, and even dry gangrene if not treated in time.

The key to successfully treating a upper arm compartment syndrome is early recognition. Abnormal pain may be the only symptom of early onset [21]. Special vigilance should be exercised in children or patients with dull pain or sedation [22]. In the diagnosis of compartment syndrome, physicians should be highly suspicious of compartment syndrome in patients with a history of related diseases if they have abnormal pain, high tension of the compartment, and passive traction pain. Compartment pressure measurements should not be relied upon [23], nor can the diagnosis be made until the limbs show late signs, such as motor palsy, paleness, or pulselessness [2].

Because of atypical injury mechanism and rare incidence, clinicians should fully recognize the fact that acute compartment syndrome can occur in the upper arm, rather than only the forearm and leg, and the increase of pressure in compartment caused by single brachial muscle is enough to cause compartment syndrome, therefore avoid serious consequences caused by missed diagnosis and misdiagnosis.

## Data Availability

The dataset supporting the conclusions of this article is included within the article.

## Abbreviation

ACS: Acute compartment syndrome
